# Extracellular vesicles and glycans: new avenue for biomarker research

**DOI:** 10.11613/BM.2024.020503

**Published:** 2024-06-15

**Authors:** Tamara Janković, Miroslava Janković

**Affiliations:** Department for Immunochemistry and Glycobiology, Institute for the Application of Nuclear Energy, INEP, University of Belgrade, Belgrade, Serbia

**Keywords:** biomarkers, extracellular vesicles, glycans, prostasomes, seminal plasma

## Abstract

The investigation of biomarkers is constantly evolving. New molecules and molecular assemblies, such as soluble and particulate complexes, emerged as biomarkers from basic research and investigation of different proteomes, genomes, and glycomes. Extracellular vesicles (EVs), and glycans, complex carbohydrates are ubiquitous in nature. The composition and structure of both reflect physiological state of paternal cells and are strikingly changed in diseases. The EV-associated glycans, alone or in combination with soluble glycans in related biological fluids, used as analytes, aim to capture full complex biomarker picture, enabling its use in different clinical settings. Bringing together EVs and glycans can help to extract meaningful data from their extreme and distinct heterogeneities for use in the real-time diagnostics. The glycans on the surface of EVs could mark their subpopulations and establish the glycosignature, the solubilisation signature and molecular patterns. They all contribute to a new way of looking at and looking for composite biomarkers.

## Graphical abstract

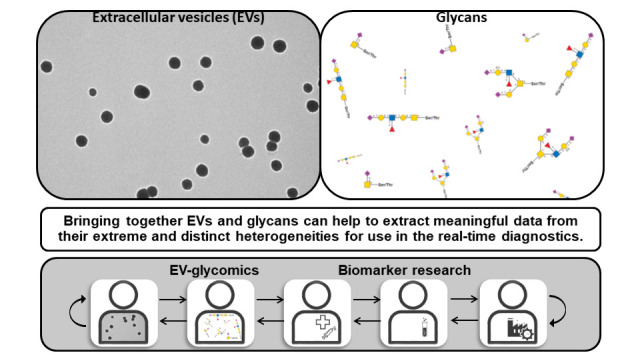

## Highlights

• Biological fluids contain nano-sized particles called extracellular vesicles

• Extracellular vesicles properties reflect the physiological and pathological state of the parent cells

• Extracellular vesicles as analytes are suitable for use in liquid biopsy-based real-time diagnostics

• Glycans, complex oligosaccharides, are distinct components of extracellular vesicles membrane and cargo

• Mapping extracellular vesicles glycans is of importance for finding new composite biomarkers

## Introduction

This review gives an overview of specific topic on extracellular vesicles (EVs) and glycans, which is focused on theoretical frame, and methodological perspectives of their possible use in clinical chemistry. Extracellular vesicles are nano-sized vesicles released by all cells (*1*). Glycans, complex carbohydrates, are also ubiquitous to all cells (*2*-*4*). Extracellular vesicles and glycans exhibit extreme heterogeneity in terms of structure, composition and function (*1*, *3*, *4*). When discovered, each category was initially neglected as a biological player, and many years passed until their crucial role in basic physiological processes was recognized (*5*). Thus, EVs are currently considered as a new mode for communication between cells, and glycans as a principle for coding and storage of biological information (*6*-*8*). Consequently, parallel investigations in these two fields have been crossed at the point of biomarkers.

Regarding biomarkers, the availability of EVs/EVs glycans *via* liquid biopsy and the possibility of multicomponent analysis are of special importance (*9*). However, due to limitations caused by intrinsic structural properties, their translation in clinic is still in infancy, awaiting the development of appropriate analytical tools for medical laboratories (*10*). This review is a contribution to the general discussion on challenges in EV-glycomics and biomarker discoveries aiming to emphasize the need for a concerted action of researchers in basic sciences, with medical biochemists, clinicians, and biotechnological companies in order to introduce innovations in the field of healthcare.

## Extracellular vesicles

### Extracellular vesicles as heterogeneous particulate analytes

Biological fluids contain nano-sized particles that share similar biophysical properties and biochemical compositions but carry distinctive clinically relevant data as analytes in medical biochemistry. Lipoproteins, well known biomarkers, are complex heterogeneous particles of lipids and proteins (*11*, *12*). Their compositions vary within their classes, high density lipoproteins (HDL) are very heterogeneous, whereas very low density lipoproteins (VLDL) and low density lipoproteins (LDL) comprise a continuum of particles of decreasing size and density (*11*, *12*).

In contrast to lipoproteins, which are non-vesicular structures having single amphipathic phospholipid (with cholesterol and apolipoproteins) outside layer and non-polar lipid core, the human body contains structures called extracellular vesicles (EVs) (*1*, *13*, *14*). They carry lipoprotein-free cholesterol; have a phospholipid bilayer (with proteins, glycoproteins) and an aqueous core. In biological fluids that are comprised of secretions from different cells and tissues, EVs are very heterogeneous and of diverse origin (*1*). Their main classes, overlapping in lipoprotein size distribution, are: exosomes of 50-150 nm, microvesicles of 100 nm-1 µm and apoptotic bodies of 50 nm-5 µm. Extracellular vesicle classes have different pathways of biogenesis: plasma membrane blebbing (apoptotic body), plasma membrane budding (microvesicles) and multivesicular body exocytosis, that is, canonical endosome pathway (exosomes) (*1*, *13*).

Accumulated experimental evidence indicates that they serves as a natural carrier of different molecules (proteins, lipids, deoxyribonucleic acid (DNA), ribonucleic acid (RNA)) from their cells/tissues of origin (*6*, *7*, *15*). In this way, they reflect the physiological and pathological state of the parent cells (*1*). Additionally, EVs are readily available from blood and urine as convenient samples for laboratory testing (*16*). All this makes them the “rising stars” in diagnostics, and they are considered as next-generation highly specific and sensitive biomarkers (*17*). It is suggested that in future, the impact of EVs on biotechnology could be as great as that of antibodies, currently the most lucrative biopharmaceutical products (*18*, *19*).

### Biogenesis of extracellular vesicles

The biogenesis of EVs is not completely understood. Microvesicles (MVs) are formed through the direct outward budding of the plasma membrane. Their biogenesis is based on a combination of plasma membrane phospholipid redistribution and coordination of actomyosin contractile machinery (*20*). Mature MVs are shed directly into the extracellular space, following tightly regulated pinching and scission processes. Small guanosine triphosphate hydrolases (GTPases) are common component of different MVs regulatory pathways (*20*).

Apoptotic bodies are released upon cell fragmentation during late phase of apoptosis. The formation of apoptotic bodies includes: membrane blebbing on the cell surface, formation of membrane protrusions and formation of vesicles (*21*). These processes are regulated by different molecular factors including: rho-associated protein kinase 1 (ROCK1), myosin-light chain kinase, and actomyosin (*21*).

Exosome biogenesis begins when cargo, internalized by inward budding of the plasma membrane, is sorted in the early endosome which further matures into a late endosome or multivesicular body (MVB) (*1*, *13*, *22*). It is rich in intraluminal vesicles (ILVs), which capture potential exosome cargoes partially originating from the trans-Golgi network and cytosol. Multivesicular bodies fuse with the plasma membrane and ILVs are secreted as exosomes. Multiple mechanisms play a role in exosome biogenesis. Those are endosomal sorting complex required for transport (ESCRT) machinery and soluble N-ethylmaleimide-sensitive factor attachment protein receptor (SNARE) proteins as well as Rab GTPases (members of rat sarcoma viruses (Ras) superfamily of small G proteins) which are involved in their secretion, but the importance of tetraspanins (TS) and lipids cannot be overlooked. Tetraspanins orchestrate cargo sorting, membrane organization and vesicle budding, while lipids influence membrane curvature and provide unique lipid composition (*13*). In addition to biogenesis, there are different parameters that were used for EVs classification (Table 1). The current recommendation is that EVs as an umbrella term should be used unless a biosynthetic pathway of EVs is experimentally confirmed (*23*, *24*).

**Table 1:** Classification of extracellular vesicles

**Factor**		**EV populations**
Origin	Plasma membrane	Ectosomes (microparticles/microvesicles)
	Endosomal system	Exosomes
Physical characteristics	Size	Small EVs (< 200 nm) Large EVs (> 200 nm)
	Density	Low density EVs Middle density EVs High density EVs
Biochemical composition	Surface markers	CD63, CD81, CD9, Flotilin-1, MHC class II
	Cargo	TSG101, Alix, HSP70 RNA, DNA
According to (23). EV – extracellular vesicles. CD - cluster of differentiation. MHC - major histocompatibility complex. TSG - tumor susceptibility gene. Alix - apoptosis linked gene 2-interacting protein X. HSP - heat shock protein. RNA - ribonucleic acid. DNA - deoxyribonucleic acid.		

Overall, EVs comprise a continuum of vesicles of different size and properties (*1*). This is even more complicated with EVs from biological fluids that comprise the secretome of different cells/tissues. Tetraspanins, which are integral membrane proteins, are considered canonical vesicular markers, but their distribution patterns are characteristic for different populations of EVs (*25*).

Experimental data obtained from EVs derived from different sources indicated various factors that contribute to their biophysical and molecular heterogeneity (*13*, *14*). Thus, there are different subpopulations of EVs which differ in respect to ultrastructure, membrane and cargo composition (*26*, *27*). Size distribution (the number within defined size categories) of EVs is also considered as possible informative indicator.

Although isolation protocols influence the shape of EVs, there is an intrinsic heterogeneity related to the presence of: single membrane or double membrane, membrane coating, filaments, electron dense area, vesicle inside vesicle, tubules (oval, small, and large), *etc.* (*26*, *28*-*30*).

Low and high density EVs resolved by gradient ultracentrifugation represent two distinct subpopulations differing further in respect to RNA, lipid and protein composition, possibly due to different biogenesis and loading (*26*). High density EVs are smaller and associated with ribosomal proteins whereas low density EVs are associated with mitochondrial proteins (*31*).

Extracellular vesicles subpopulations can also be divided according to the presence of molecules such as classical exosomal markers (tetraspanins CD63, CD81, CD9, tumor susceptibility gene 101 (TSG101), apoptosis linked gene 2-interacting protein X (Alix)) or other specific proteins (MHC class II, HSP70, Flotilin-1) as well as RNA (microRNAs (miRNAs), long noncoding RNAs (lncRNAs), circular RNAs (circRNAs), small nucleolar RNA (snoRNAs), small nuclear RNAs (snRNAs), transfer RNA (tRNAs), ribosomal RNAs (rRNAs), and piwi-interacting RNAs (piRNAs)) and DNA (single, double-stranded, genomic or mitochondrial) cargo (*26*, *32*, *33*). The difference in EVs functions that can be related to their origin (organ/cell) and subsequently with composition/cargo are also considered as sources of heterogeneity.

### Extracellular vesicles application in liquid biopsy

In contrast to conventional biopsy, which is performed by surgery or needle aspiration, liquid biopsy is a less invasive technique and is based on the sampling of biological fluids (blood, urine, cerebrospinal fluid, tears and milk). The liquid bioptate contains various products of different tissues in one place, and sampling can be repeated over time enabling clinicians to overcome limitations of classical solid biopsy. The link between tissues and biological fluid, predominantly peripheral blood as the most commonly used sample for laboratory analysis, is established by analysis of cellular and non-cellular (lipids, metabolites, proteins/glycoproteins, *etc.*) liquid biopsy analytes. Non-cellular analytes also include EVs, which currently are receiving growing attention in the field of liquid biopsy, as their analysis is considered advantageous over the analysis of other analytes (*9*, *34*, *35*).

Peripheral blood is an easily available source of EVs, with their number estimated to be 10^11^ particles/mL of blood (*34*). Other biological fluids are also a rich source of EVs, but estimation of EVs concentrations is prone to error due to the methods used (detection of any particles, not specifically EVs) (*34*). In addition, they are biologically stable (due to the lipid coat) and have long half-lives in circulation (*36*). As a part of secretome, they allow monitoring changes in parental cells during pathological and physiological processes. Extracellular vesicles can be considered as biomarkers themselves (by number and size). Moreover, they represent a source of known/specific biomarkers being concentrated in this particulate analyte. Taken together, they are suitable for both single of multiplexed analysis.

The purity of EVs preparations is critical for their reliable use in liquid biopsy-based diagnostics. However, there are many obstacles to standardize EVs preparations and no single method currently used is ideal (*37*, *38*). Differential centrifugation and ultracentrifugation/density gradient centrifugation and size exclusion chromatography (SEC) are mostly used (Table 2). The choice of method is suggested to be aim/analyte dependent (*39*, *40*). To reduce contamination, the combination of methods is preferable.

**Table 2:** Isolation techniques for extracellular vesicles

**Techniques**	**Advantages**	**Disadvantages**
Ultracentrifugation	Scalable for both small and large volumes Cost-effective	Risk of EVs aggregation Co-isolation of HDL Time-consuming procedure
Density gradient centrifugation	Pure preparation	Low yield Time-consuming procedure
Size exclusion chromatography	Maintain the integrity of EVs Refined and controlled process	Sample dilution Variable recovery Co-isolation of chylomicrons and VLDL
Ultrafiltration	Simple procedure No limitations on sample volume	Filter plugging Contamination with proteins
Polymer-based precipitation	Low cost Simplicity of the procedure	Protein contaminants Retention of chemicals or polymers Long processing time
Immuno/glyco affinity-based capture	Enrichment of EV subpopulations High specificity Targeting specific surface markers/glycans	Some EVs subpopulations have unknown specific markers Potential cross-reactivity Cost
Microfluidic technologies	Reduced sample volume High throughput Rapid isolation	Limited scalability Cost
According to (35-37). EV – extracellular vesicles. HDL - high density lipoproteins. VLDL - very low density lipoprotein.		

Extracellular vesicles in isolate can be monitored and characterized using transmission or scanning electron microscopy (*41*). In addition, estimation of EVs number and size in corresponding isolates are part of standard procedure. For this purpose, nanoparticle tracking analysis (NTA) or dynamic light scattering (DLS) is used (*42*). Antibodies to EV specific markers/biomarkers can also be used in different experimental formats such as western blot, flow cytometry, *etc* (*35*).

Preanalytical procedures have significant to variable or no impact on EVs isolation (Table 3) (*23*, *43*, *44*). There is no simple solution but some generalizations are given as directions.

**Table 3:** Preanalytical variables influencing extracellular vesicles analysis

**Factor**	**Preanalytical variable**
Characteristics of biological fluid	Viscosity Contaminants specific to biological fluid: LDL, VLDL, HDL (serum/plasma) fat containing vesicles (milk) Tamm-Horsfall protein (urine) surfactant (bronchoalveolar lavage)
Sample handling	Sample handling time Storage Transportation Collection volume Type of container Choice of anticoagulant Degree of hemolysis Platelet and lipoproteins depletion Protocol for centrifugation
Patient features	Age Sex Pre/postprandial status Circadian rhythm Exercise Diet Body mass index Infections Medications
According to (23,43,44). LDL - low density lipoproteins. VLDL - very low density lipoproteins. HDL - high density lipoproteins.	

The contaminants present in different biological fluids will be isolated to various degrees with EVs, and specific precautions to separate EVs from these components may be required (*23*, *43*). Regarding sample handling, serum is less affected than plasma due to significant influence of anticoagulants (*23*, *43*, *44*). Serum and citrate plasma contain increased numbers of platelet-derived EVs compared to acid citrate dextrose (ACD) and ethylenediaminetetraacetic acid (EDTA) plasma samples. Platelet elimination is also very important due to the influence of the transportation of samples, the times from collection to isolation and centrifugation protocols (*43*, *44*).

The preferable option is to centrifuge the samples immediately (at the site of collection) or wait 24 hours before centrifugation to avoid removal of small EVs with cells (*23*, *43*).

Extracellular vesicles may be lost upon storage by adhering to the surfaces of storage containers. Isotonic buffers are recommended for storing EVs to prevent pH shifts during storage, freezing and thawing (*23*, *43*). Repeated freeze-thaw cycles should be avoided to prevent EV aggregation. Although the storage temperature and freeze-thaw cycles are not as important, data obtained from frozen biobank samples should not be compared to fresh samples (*23*, *43*, *44*).

### Extracellular vesicles as *in vitro* diagnostic tools

Although faced with general technical problems regarding assay performance (low reproducibility due to lack of standardization) that influence the progress and approval of these tests in clinical use, there has been a lot of work on EVs as analytes (*17*, *45*, *46*). Analysis of EVs in blood can encompass those originating from normal cells, tumor stroma, or tumorous cells. As for EVs components, analysis of RNA, DNA, and proteins can be performed on the same particle. Thus, EVs contain different types of RNA: miRNA, lncRNAs, circRNAs, snoRNAs, snRNAs, tRNAs, rRNAs, piRNAs (*17*, *47*-*49*). Regarding EV-associated DNA, it may carry different mutations, but typically represents the entire genome without fragmentation, as is often observed in circulating tumor DNA (ctDNA). In summary, EVs RNAs (miRNA, lncRNA, mRNA) are much more studied than DNA (mutated) or proteins in terms of clinical diagnosis, prognosis, or therapeutic monitoring, especially in cancer (breast, lung, hepatocellular, ovarian, bladder, colorectal prostate, melanoma) (*9*, *17*, *34*, *35*). Taking advantage of EVs structure/functions, their application in multicomponent diagnostics is gaining attention. Compared to current approaches, the aim is to design a combination of high-throughput analyses and deep learning interpretation to improve the applicability of individual assays (*50*).

Companies which are involved in the EVs-related services and products were recently reviewed (*17*). Although, the manufacturing of EVs diagnostics is still in its early developmental stages, notable progress has been made. Exosome Diagnostics (Waltham, USA) has successfully produced the commercially available ExoDx test, which received Food and Drug Administration (FDA) breakthrough device designation in 2019. This urine-derived EVs-based test helps avoid unnecessary biopsies in older patients with prostate-specific antigen (PSA) concentrations in the grey zone, indicating suspicion of prostate cancer. Commercially available miR Sentinel test, which received FDA breakthrough device designation in 2020, has been introduced by miR Scientific (New York, USA). This test focuses on prostate cancer detection based on sncRNA derived from urinary EVs. Additionally, a salivary-based EVs miRNA biomarker, miR-185, for the diagnosis and treatment of oral cancer was developed.

In addition, there are many companies and academic institutions which have been actively engaged in the assessment of EVs biomarkers using different technological approaches (*17*). Thus, Exosome Plus (Gyeonggi-do, South Korea) has introduced the ExoThera platform, focusing on EVs-based therapies, and has plans to develop a liquid biopsy platform for 11 major cancer types. Mercy Bionalytics (Waltham, USA) created the Halo test for early detection of ovarian and lung cancers. Craif (Tokyo, Japan) developed the unique method for extracting EVs using microfluidic nanowire devices and cellulose nanofibers. Cutting-edge analytical and measurement systems are aimed to improve challenging EVs-associated microRNA and other trace nucleic acid analyses.

In the arena of clinical trials for EVs diagnostics, numerous studies, both completed and ongoing, have focused on various medical conditions, including cancer, neurological diseases, and respiratory and heart dysfunction (*17*). EVs from blood/plasma or urine are mostly used.

## Glycans

### Basic glycobiology concepts

Glycans are complex carbohydrates which can be covalently linked to proteins or lipids to form glycoproteins/proteoglycans and glycolipids (*3*, *4*). Recently, it was shown that glycans can be also attached to small non-coding RNA (*51*). There are several classes of glycans: glycoproteins/proteoglycans, glycosphingolipids, glycosaminoglycans, and free oligosaccharides (*3*, *4*).

Protein glycosylation is a co- and post-translational modification (*3*, *4*). N-glycosylation begins in the endoplasmic reticulum (ER) and continues in the Golgi apparatus, while O-glycosylation primarily occurs in the Golgi apparatus. Thus, glycosylation steps form an integral part of the secretory machinery of the cell. Glycans are assembled without a template and the resulting structure is not unique.

In addition to the common N- (linkage to the nitrogen atom of asparagine within a specific amino acid sequence motif asparagine-X-serine/threonine, in which “X” is any amino acid except for proline (Pro)) and O- (linkage to serine (Ser) or threonine (Thr)) glycosylation, there are also P-glycosylation (linkage to serine or threonine *via* phosphodiesters), S-glycosylation (linkage to cysteine), C-glycosylation (acarbon-carbon bond at an anomeric carbon of carbohydrates) and glypiation (linkage to conserved glycosylphosphatidylinositol (GPI)) (*52*).

N-glycans share a common core sequence (two N-acetylglucosamine (GlcNAc) residues and three mannose (Man) residues), and are classified into three types: oligomannose, in which only Man residues extend the core; complex, in which “antennae” initiated by GlcNAc extend the core; and hybrid, in which Man extends the Manα1-6 arm of the core and one or two GlcNAc-initiated antennae extend the Manα1-3 arm (*3*, *4*).

As for O-glycans, the most common sugars linked to Ser/Thr are N-acetylgalactosamine (GalNAc) and GlcNAc. N-acetylgalactosamine O-linked to Ser/Thr is the initiating sugar of O-GalNAc glycans and is usually extended to form one of four common core structures which can subsequently be extended to give a mature linear or branched O-GalNAc glycan (*3*, *4*). Addition of GlcNAc to Ser or Thr does not typically occur in the Golgi apparatus and is not extended. This O-glycan is specifically found on nuclear, mitochondrial and cytoplasmic glycoproteins (*3*, *4*).

The additional sources of heterogeneity are variation in monosaccharide sequence (most common are hexopyranose, 6-deoxy hexoses, acetamino sugars, sialic acid), carbohydrate isomers, and modifications, as well as branching patterns of oligosaccharide chains. A single glycoprotein can have both N- and O-glycans as well as more than one N- or O-linked glycan. Microheterogeneity as variation between individual molecules, usually at the terminal position in oligosaccharide chains, is intrinsic to glycoproteins (*3*, *4*). It results in the existence of different glycoforms of a particular molecule. The composition of these glycoforms is known to change under normal physiological and pathological conditions (*53*-*55*). This can influence molecule stability, its antigenic and binding properties, and change its intracellular transport, secretion as well as biological function. It could indirectly be a reflection of changes in the activity of glycosylation enzymes and the availability of related substrates at different stages of glycoprotein processing steps (*3*, *4*).

Glycoproteins as analytes are heterogeneous and no reference materials for glycoprotein biomarkers are available (*56*, *57*). This is due to their structural properties since they can exists as different splicing variants, isoforms, degradation products, oligomers or in complex with various ligands (*3*, *4*). All this can influence standardization, comparability and analytical performances of laboratory assays (*56*, *57*). Serum proteins are mostly glycosylated and known as readily available biomarkers, but clinical laboratory analysis seldom includes analyses of serum protein glycoforms (*58*, *59*).

### Glycan analysis

Glycome, the entire set of glycans of an organism (or a the cell), is thought to be a rich source of biomarkers (*60*). Glycomics aims to define the complete repertoire of glycans produced by the cell or organism under specified conditions of time, location and environment (*61*, *62*). Glycoproteomics aims to determine glycome on the cellular proteome. For glycome analysis, various analytical techniques, instrumentation, and bioinformatics tools were developed (*61*-*63*). High throughput glycomics relies on different technologies such as: liquid chromatography (LC), capillary electrophoresis (CE), mass spectrometry (MS), and lectin microarrays (*63*). Depending on what level of complexity should be described, glycoprofiling, glycan class characterization, and full structural analysis can be performed (*4*, *61*, *62*). Glycoprofiling as the starting point, represent one-dimensional separation of mixed glycans released from their scaffolds, and gives essential overview of glycan structures. Glycan class characterization implies separation of glycan mixtures into glycan types, and can provide relative quantitation of different glycan classes. Full structural analysis involves: determination of the monosaccharide sequence and modifications, anomericity, and linkage of the glycans (*61*, *62*).

Glycomic analysis can results in description of glycan structures as well as related quantitative information (*4*, *61*-*63*). The related biomarkers can be based solely on glycan compositions and they are suitable for disease detection. In addition, finding specific glycans (isomer separation, protein-specific and site-specific glycosylation) associated with individual proteins and lipids which are expressed in a specific place/time may improve clinical utility (insight into tissue location and progression/severity of disease) of a particular biomarker (*4*, *61*-*63*).

In general, it is shown that glycosylation traits rather than single glycans could serve as a basis for the identification of disease-specific glycan signature and the development of a specific diagnostic tests (*4*, *61*-*63*).

Thus, precise determination of the relative abundance of individual site-specific glycan structures is the key to identifying glycan biomarkers (*4*, *61*-*63*). Development of the appropriate analysis tools is expected to be exploited not only for the early diagnosis but also as an adjunct parameter of disease phenotype.

## Biomarkers

### Glycans of extracellular vesicles as biomarkers

Glycoproteins and glycolipids reside on the extracellular surface of the plasma membrane, are present as soluble molecules in serum and other biological fluids, as insoluble molecules in the extracellular matrix or on extracellular particles including EVs. Glycoproteins can be integral parts of the EV membrane or exist as EVs membrane-associated molecules *via* different ligands, both contributing to the EVs corona, analogously to the cell glycocalyx (*64*). Glycans which act as a molecular bar code on cell surface could also be considered as a possible factor to differentiate between EVs sharing similar biophysical properties (*8*, *65*, *66*).

**Figure 1:**
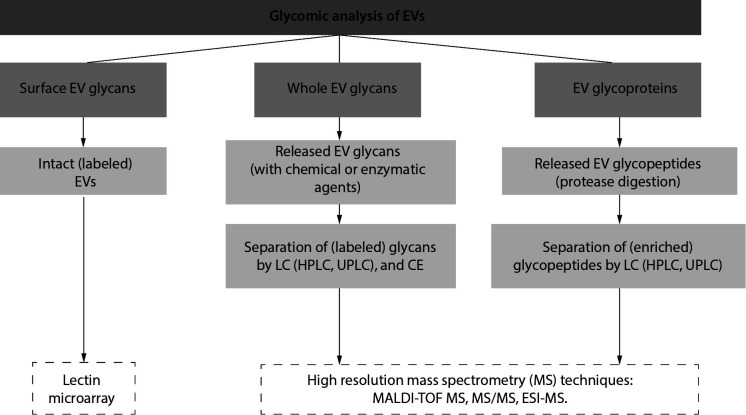
Shematic representation of glycomic analysis of extracellular vesicles (*61*-*63*). EVs - extracellular vesicles. LC - liquid chromatography. HPLC - high-performance liquid chromatography. UPLC - ultra-performance liquid chromatography. CE - capillary electrophoresis. MALDI-TOF MS - matrix-assisted laser desorption-ionisation-time of flight mass spectrometry. ESI-MS - electrospray ionization mass spectrometry.

Approaches to study EVs glycans and glycans themselves are analogous (Figure 1). A pioneering study of EVs glycome (using lectin microarrays) from T-cell-derived EVs revealed surface enrichment (compared to originating cell) of polylactosamine and alpha 2-6 linked sialic acid residues, complex type N-glycans and high mannose structures (*67*, *68*). This glycan signature was also found to be conserved across various cellular sources (*68*). Analyses of the glycan composition of EVs have emphasized glycome changes in various diseases states (prostate, ovarian, colorectal, and lung cancer, galactosemia, autosomal dominant polycystic kidney disease, *etc.*), thus indicating biomarker potential of EVs glycans (*66*, *69*-*71*). As for cancer, the most studied condition regarding EV pathobiology, accumulated experimental evidence revealed increased terminal sialylation, core fucosylated N-glycans, N-glycans branching, truncated O-glycans, and increased O-GlcNAcylation (*45*, *66*, *70*).

In general, the distribution of an individual glycoprotein is determined by its structure and also depends on the expression and availability of the molecules responsible for its trafficking or its binding partners. Based on this, the new approach in the search for biomarkers is based on the binding properties of glycoprotein as overlooked determinates of heterogeneous analytes (*72*, *73*). They could be used as targets for the designation of novel analytical approaches aimed to establish glycosignature, the solubilization signature and molecular patterns as possible EVs-associated biomarkers (Table 4). On the other hand, the binding properties of glycoproteins could be relevant for other clinical purposes, since glycan-mediated interactions contribute the functional properties of EVs (*74*-*76*).

**Table 4:** Molecular and analytical features qualifying extracellular vesicles/glycans for composite biomarker candidates

**Parameter**		**Biomarker potential**
Glycosignature	1. EV subpopulations separated by lectin-affinity chromatography. 2. Lectin-reactivity annotated to separated EV subpopulations (combined with TS, enzymes, and other selected surface-associated molecules).	Ratio of defined EV subpopulations in biological fluids in health and disease. Changes in the surface composition of defined EVs subpopulations in health and disease.
Solubilization signature	Lectin-reactivity annotated to the detergent-resistant EVs membrane domains (combined with TS, enzymes, and other selected surface-associated molecules).	Changes in the distribution of distinct molecules in the detergent-resistant membrane domains in health and disease.
	Molecular pattern of the solubilized EVs (detergent-sensitive membrane domains).	Changes in the molecular pattern (composition and lectin-reactivity) of the solubilized EVs (detergent-sensitive membrane domains) in health and disease.
EVs as carriers	1. EVs isolated by ultracentrifugation and size exclusion chromatography. 2. Membrane associated enzymes (such as GGT) or other membrane molecules annotated to EVs.	Changes in the ratio of GGT fraction across soluble (free and high molecular mass enzyme form) and EVs-associated form in health and disease.
EV – extracellular vesicles. TS – tetraspanins. GGT - gamma-glutamyl transferase.		

### Glycosignature of prostasomes, extracellular vesicles from human seminal plasma

Human seminal plasma (hSP) is a composite fluid produced by male accessory reproductive glands and the epididymis (*77*). It is extremely rich in EVs, the most abundant of which are those created by epithelial cells of the prostate, known as prostasomes (*30*, *78*). They show great heterogeneity in terms of size, internal morphology, and molecular composition, and the mechanism of their biogenesis corresponds to the biogenesis of exosomes. Thus, prostasomes have been reported to differ in size (50-200 nm), shape (elongated, round, pear-shaped), presence/absence of protrusions, and density (dark and light vesicles) (*30*). The membrane of prostasomes is rich in diverse bioactive molecules (enzymes, receptors, cytokines) and the property distinguishing them from EVs from other sources is their distinctive lipid composition and organization. The composition is characterized by a high concentration of cholesterol and sphingomyelin, resulting in the formation of lipid rafts (*79*, *80*). Prostasomes also have functional Janus face, *i.e.* their pluripotency favors the normal reproductive process and malignant prostate growth (*81*, *82*). It is supposed that the ability of prostasomes to promote fertility (increase sperm motility, immunosuppressive properties) can also promote the survival and progression of prostate cancer cells (mediated by phosphorylation enzymes and metalloenzymes, angiotensin-converting enzyme (ACE), tissue factor, and chromogranin A) (*83*).

Prostasomes are defined according to the so-called prostasomal signature. It is exemplified by three 90-150 kDa protein bands identified as aminopeptidase N (CD13), dipeptidyl peptidase IV (CD26), and neprilysin/enkephalinase (CD10) (*84*).

Annotation of lectin-reactivity (suggesting main surface glycan traits) to prostasomes (populations/subpopulations) was based on the results of lectin-affinity methods designed and performed to enable characterizing prostasomal surface. Density, nature, and mode of recruitment of glycans into organized complexes, such as corona, in particular, the TS web (a network of TS and their partner proteins that facilitate cellular interactions), or galectin-glycoprotein lattice influence presentation and accessibility of possible lectin ligands (*85*-*87*). Lectin-reactivity in association with selected TS, canonical EVs markers, additionally annotated prostasomal heterogeneity, and point out discrete differences among vesicles populations (*86*). Sialylated and mannosylated glycans, as deduced from the binding of WGA (wheat germ agglutinin) and concanavalin A (Con A) agglutinin, both mark prostasomal populations that additionally differentiate in terms of the activity of the membrane-associated enzyme: gamma-glutamyl transferase (GGT) and alkaline phosphatase (ALP).

The molecular disposition of sialylated and mannosylated glycans and the TS: CD63, CD9, and CD81 on detergent-sensitive /detergent-resistant membrane domains were also established as potential referent parameters for comparison (*88*). The results obtained indicated that CD9, galectin-3 (gal-3), and WGA - reactive glycans were distributed in detergent-resistant domains, while CD63, GGT, and Con A-reactive glycans were located in detergent-sensitive domains.

Collectively, these studies identified four to six distinct glyco-signatures in intact and solubilized prostasomes. When comparing prostasomes from normozoospermic and oligozoospermic individuals, subtle variations were observed in these populations regarding their associated ALP-activity and the distribution of integral EVs membrane proteins (*85*, *86*). Furthermore, WGA-separated prostasomal populations were found to be similar in prostasomal preparations of normozoospermic men and oligozoospermic men, while Con A-separated ones were different. Defining specific composite biomarker: co-distribution of sialylated and mannosylated glycans, selected TS and enzymes as well as gal-3, represents a starting point and a necessary piece of data that would be used as a reference for various types of comparative analyses related to conditions important for male reproductive health.

### Glycan-associated patterns across EVs- and soluble subproteomes of hSP

The investigation of EVs is important from the perspective of their role as carriers of different biomarkers. This led to the *in vivo* appearance of distinct low molecular mass biomarkers in the high molecular mass fraction of different biological fluids. In view of this, an understanding of the analytical and biomarker potential of molecular patterns of transmembrane/membrane-bound proteins could be of special interest. Therefore, it is possible that, depending on the mode of release, hydrophilic species (without membrane domain) or hydrophobic species prone to autoaggregation or forming complexes with lipid moieties could be present as higher molecular mass forms in the soluble subproteome of a distinct biological fluid (*89*). However, some fraction corresponding to integral membrane proteins residing on the EV membrane itself could also be co-distributed.

One of such protein of interest as a biomarkers is GGT which is expressed in almost all cells. It is a membrane-bound enzyme, known as a marker of gastrointestinal diseases, and is being used regularly in clinical chemistry (*90*). It is also considered as a EVs marker. There are four fractions of GGT that differ in molecular mass: free GGT (f-GGT) of 70 kDa, small GGT (s-GGT) of 250 kDa, medium GGT (m-GGT) of 1000 kDa, and big GGT (b-GGT) of 2000 kDa (*91*, *92*). The biogenesis and physical and molecular properties of m-GGT, s-GGT and f-GGT fractions are not fully understood. It is suggested that they might arise from progressive modifications (protein cleavage) of the b-GGT fraction (*93*).

Higher GGT concentration in serum is found in different pathologies (*90*). Recently, the importance of the existence of different GGT forms regarding its biomarker potential was suggested. This is based on fractional GGT patterns, that is, changes in the ratio of particular fractions, usually b-GGT and s-GGT, under normal versus pathological conditions (*93*-*95*). Furthermore, b-GGT is thought to be heterogeneous and can comprise different particulate/membrane structures, or macromolecular protein complexes (*92*).

Based on data highlighting the heterogeneity of b-GGT and its extremely high concentration in hSP (*96*) which is also a rich source of prostasomes, the heterogeneity of GGT was studied in a new way (*97*). Glycobiochemical characterizations of molecular patterns that contain GGT were examined as a possible target for increasing its clinical utility. They were established by annotation of the main contributing glycoproteins in the soluble subproteome of hSP or seminal prostasomes to the related GGT molecular mass forms. Molecular patterns qualitatively describe a range of proteins and glycoproteins, some being consistently present while the presence of others may vary among samples. In general, WGA-reactive sialylated mucin-like glycans were associated with GGT in the soluble subproteome of hSP, whereas Con A-reactive mannosylated glycans were characteristic for particular prostasome subproteome. The results obtained provided insights into the presence and distribution of b-GGT and EVs/soluble glycans, collectively forming possible composite biomarkers.

In summary, GGT activity in unfractionated hSP, which was found to be distributed in soluble and prostasomal subproteomes, appears to be present in diverse biological environments. That is why measuring of GGT activity in this sample and, analogously, in other biological fluids may be influenced by the most abundant form or by interference/favoritism due to distinct spatial molecular arrangements of some of them as previously observed (*92*).

## Conclusion

Achievements in basic research involving EVs and glycans open the road to their multiple application in biomedicine. Biotechnological companies developed various *in vitro* solutions designated to use the contents of EVs as biomarkers. Having in mind that there are various levels of disease complexity, it should be expected that EVs and glycans *i.e.* EVs glycan analyses could become an integral part of human laboratory medicine. Challenges still remain in the development of more standardized purification and analytical procedures as well as interpretation protocols.

## Data Availability

No data was generated during this study, so data sharing statement is not applicable to this article.
